# Antifungal Activity of Benzoquinones Produced by *Tribolium castaneum* in Maize-Associated Fungi

**DOI:** 10.3390/insects13100868

**Published:** 2022-09-24

**Authors:** Sónia Duarte, Ana Magro, Joanna Tomás, Carolina Hilário, Ricardo Boavida Ferreira, Maria Otília Carvalho

**Affiliations:** 1 LEAF-Linking Landscape, Environment, Agriculture and Food, TERRA—Laboratory for Sustainable Land Use and Ecosystem Services, Universidade de Lisboa, Instituto Superior de Agronomia, Tapada da Ajuda, 1349-017 Lisboa, Portugal; 2 Instituto Superior de Agronomia, Universidade de Lisboa, Tapada da Ajuda, 1349-017 Lisboa, Portugal

**Keywords:** benzoquinones, stored maize fungi, *Tribolium castaneum*

## Abstract

**Simple Summary:**

The environmental conditions selected to store food products can be favorable to the development of different biotic degradation agents, such as insects and fungi. The interactions between these two groups of organisms may be multiple, and not restricted to antagonistic relationships. Competition may arise between stored products associated fungi, and insects, as they often thrive for the same food sources. Adults of *Tribolium castaneum* insects, a major stored food-product pest worldwide, produce benzoquinones, which are released as chemical defenses against other organisms. This study evaluated the effect of these substances on the development of six maize-associated fungal species: *Aspergillus flavus*, *A. fumigatus*, *A. niger*, *Fusarium* sp., *Penicillium* sp., and *Trichoderma* sp. The results obtained showed that all the fungi tested are susceptible to the benzoquinones produced by *T. castaneum*, but the level of sensivity is species specific, with most of the fungi showing a delayed growth. The contact of *Penicillium* sp. with a mixture of two benzoquinones, produced by *T. castaneum*, was deadly. Revealing the nature of the relationship between some fungal species and adults of *T. castaneum* can be a step forward for a better management in the preservation of stored food products.

**Abstract:**

*Tribolium castaneum* (Herbst) adults produce 1,4-benzoquinone (BQ), methyl-1,4-benzoquinone (MBQ), and ethyl-1,4-benzoquinone (EBQ). These components are chemical defenses used as repellents and irritants, and BQ has a negative impact on the growth of some fungal species. In this work, the inhibitory and/or lethal effects of these benzoquinones on the development of six fungi identified in maize, namely *Aspergillus flavus*, *A. fumigatus*, *A. niger*, *Fusarium* sp., *Penicillium* sp., and *Trichoderma* sp., were evaluated. Ten-day-long disk diffusion trials were performed using benzoquinones. The experiments simulated the activity of BQ (B1) or “EBQ + MBQ” (B2) released by 40-day-old insect adults (n = 200), considering a total average release of 45 µg per adult. Inhibition halos imposed by benzoquinones on fungal growth showed a significant effect when compared with the controls (water and solvent). Mycelial growth was decreased for all fungi, with the level of response depending on the fungal species. B1 and B2 displayed an inhibitory effect against all fungi, but *Trichoderma* sp. and *A. niger* showed rapid recoveries. B2 showed a lethal effect on *Penicillium* sp. The inhibitory and lethal activities of benzoquinones released by *T. castaneum* adults may contribute to regulate fungal growth, and understanding their interaction is important to develop innovative control strategies.

## 1. Introduction

*Tribolium castaneum* (Herbst) is a member of the most species-rich eukaryotic order (Coleoptera). It is considered a model organism for the study of insect development and an important pest of stored agricultural products [1,2]. *T*. *castaneum* cytochrome P450 proteins (CYPs) are assumed to be involved in the metabolic detoxification against plant allelochemicals and toxicants, and several are insecticide resistance genes, explaining why this species exhibits resistance to almost all insecticide classes [1,3,4,5,6,7].

*T. castaneum* has evolved the ability to interact with a wide diversity of chemical environments, as shown by its wide variety of odorant and gustatory receptors, as well as cytochrome P450 and detoxification enzymes [8]. The adults release a mixture of antimicrobial compounds composed of 1,4-benzoquinone (BQ), methyl-1,4-benzoquinone (MBQ), and ethyl-1,4-benzoquinone (EBQ) [9,10]. Some researchers affirm that DNA damage caused by benzoquinones may cause cancer in people, but the International Agency for Research on Cancer (IARC) officially stated that no epidemiological data are available on the carcinogenicity of BQ. There “is inadequate evidence in experimental animals for the carcinogenicity of 1,4-benzoquinone, which consequently is not classifiable as to its carcinogenicity to humans” [11]. Benzoquinones may provide an antimicrobial coating to the cuticle of the *T. castaneum* [12,13]. EBQ and MBQ are defensive secretions used as repellents and irritants acting against potential predators, parasitoids, and microorganisms such as fungi [14,15,16,17,18]. *T. castaneum* shows higher resistance to *Beauveria bassiana* (Bals.-Criv.) Vuill. when compared to other insects species [13,19,20,21]. Some studies revealed that benzoquinones secreted by *T. castaneum* confer resistance to two generalist ascomycete entomopathogens: *B. bassiana* and *Metarhizium anisopliae* (Metchnikoff) Sorokin, as the conidia from the two fungal species did not germinate on the adult body surface [20,22].

Benzoquinones are produced by the adults of *T. castaneum* and their amount varies with insect densities, increasing with the number of individuals, and also with time but in a nonlinear way [23,24]. High levels of benzoquinones can be associated with strong densities of *T. castaneum* to act as a repellent to larvae and adults [23,25,26] or because the production of the aggregation pheromone decreases during adulthood [26].

The interactions between *T. castaneum* on one hand and fungal species associated with stored products and mycotoxin producers on the other are still poorly understood. There are studies indicating that *T. castaneum* may infest the commodities, spreading pre-existing contaminations of *Aspergillus flavus* (Link) (some strains of which can produce aflatoxins) that represent a serious threat to human and animal health after ingestion of the contaminated foods [27,28].

*T. castaneum* may also play an important role in the regulation of fungal and bacterial populations in the stored product ecosystems and may also influence the insect populations because they may exhibit predatory and cannibalistic behavior [20,29,30,31].

Considering that *T. castaneum* is one of the most important key pests of stored milled grain and generally shows resistance to chemical control and entomopathogenic fungi, the present work intends to understand if *T. castaneum* benzoquinones have an antifungal activity on fungi usually associated with maize, such as *Aspergillus flavus*, *A. fumigatus* Fresenius, *A. niger* van Tieghem, *Fusarium* sp., *Penicillium* sp., and *Trichoderma* sp.

## 2. Materials and Methods

### 2.1. Benzoquinones

Three synthetic benzoquinones were used: 1,4-benzoquinone (BQ; Sigma Aldrich, Germany), referred to as B1; and ethyl-1,4-benzoquinone (EBQ; Chemspace, Latvia) plus methyl-1,4-benzoquinone (MBQ; Sigma Aldrich, Germany) in the proportion referred in the literature [8], referred to as B2.

The total average amount of benzoquinones present in a single 40-day-old *T. castaneum* adult (45 μg of B1) was considered for the concentration calculations [8]. This value corresponds to 27 μg of EBQ and 18 μg of MBQ for B2 [8]. These calculations were made to simulate the benzoquinones released by 200 adults of *T. castaneum* after 40 days from their eclosion, using 30 μL of the solvent dimethyl sulfoxide (DMSO) (Table 1).

The solvent was selected after preliminary assays comparing the effect of methanol, ethanol [29,31] and DMSO [29,32] on fungal development. Only DMSO in the amount selected was shown to have no interference in fungal development.

The benzoquinones were tested against fungi, using the disk diffusion assay method [33], further explained in Section 2.3.

### 2.2. Fungal Species

The fungi belonging to the species *Aspergillus flavus*, *A. niger*, and to the genera *Fusarium* Link, *Penicillium* Link, and *Trichoderma* Persoon, were previously isolated from maize grains and maintained at 4 °C in the collection of the laboratory of stored products mycology, at the Instituto Superior de Agronomia, University of Lisbon, Portugal. *A. fumigatus* was also isolated from maize samples and stored under identical conditions.

Suspensions of conidia from the above-mentioned fungi were prepared on potato dextrose agar (PDA) plates, and grown for eight days, by rubbing the spore-containing surface with a curved needle. After filtering through a 60-μm mesh sieve to remove debris, enough sterile distilled water was added to prepare a spore suspension that reached the previously defined conidia concentrations, based on cell counts using a haemacytometer. For the disk diffusion assays, the conidia concentrations used were established between 10^6^ and 10^8^ conidia/mL, according to previous studies [34,35] (Table 2).

### 2.3. Disk Diffusion Assays

Disk diffusion assays were performed on Petri dishes according to a previously adapted defined methodology [34]. The fungal spore inoculum (500 μL) from each species was homogeneously dispersed with 10 sterile glass spheres onto PDA plates.

Sterile paper disks, 0.9 cm in diameter, were impregnated with 30 μL of B1 or B2, allowed to air dry, and placed face down on the inoculated agar surface. For these assays, two controls were defined: one containing fungal spore inoculum only, the other containing the filter papers impregnated with 30 μL of the solvent DMSO. Ten replicates were set for assay.

The plate assays were maintained in a climatic chamber at 28 ± 2 °C and 70 ± 5 % relative humidity. The inhibition halos were measured daily for 10 days. This measurement was made in two perpendicular directions, and the average diameter was considered.

### 2.4. Evaluation of Inhibitory/Lethal Activities

**Figure 1:**
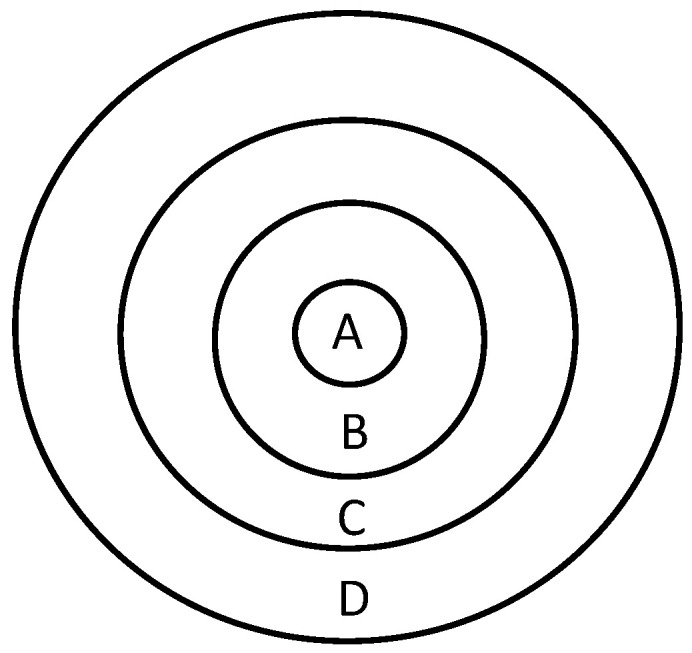
Schematic representation of the different positions from which fungal mycelium was collected for the evaluation of the fungistatic or fungicidal effects of benzoquinones. A—filter paper disk impregnated with benzoquinones, B—central zone, C—transition zone, and D—peripheral zone.

To assess the inhibitory or lethal effects of benzoquinones on fungal development, the following tests were designed. Disks (5 mm diameter) containing each fungus mycelium from the disk diffusion assays exhibiting inhibition halos were taken from three different colony positions, namely a central zone, a transition zone, and a peripheral zone, relative to the impregnated disk placement in the Petri dish (Figure 1). The evaluation of this trial was done by the attribution of two categories: 0 (the fungus did not grow) and 1 (the fungus grew).

The disks collected in previous assays (Section 2.3) were placed on Petri dishes containing PDA and incubated in a climatic chamber maintained at 28 ± 2 °C and 70 ± 5% relative humidity. After five days, the growth of the fungus was determined. Three replicates of each zone per fungus were done.

### 2.5. Statistical Analyses

After the normality test of Shapiro–Wilk and the variances test of homogeneity of Bartlett, the data were not considered to be normal (for *p* < 0.05). Therefore, to evaluate the existence of significant differences among the assays performed (fungus control, solvent control, B1 and B2) for each fungal species, a Kruskall–Wallis test was applied, followed by Wilcoxon multiple comparison tests when the Kruskall–Wallis results were considered significant (for *p* < 0.05). These analyses were done with RStudio [36] and R-3.1.2.

## 3. Results

### 3.1. Disk Diffusion Assays

There were no significant differences in fungal development for the control trials, the fungal spore inoculum only or with the solvent control (i.e., DMSO). No inhibition halos were formed in either case, indicating the absence of any fungal growth inhibition. However, fungal growth in the tests involving benzoquinones (B1 and B2) showed significant differences from the controls (*A. flavus*: χ^2^ = 110.4, *p* < 0.001; *A. fumigatus*: χ^2^ = 127.2, *p* < 0.001; *A. niger*: χ^2^= 69.8, *p* < 0.001; *Fusarium* sp.: χ^2^ = 158.9, *p* < 0.001; *Penicillium* sp.: χ^2^= 342.6, *p* < 0.001; *Trichoderma* sp.: χ^2^= 44.2, *p* < 0.001). Moreover, no overall significant differences were observed between the B1 and B2 treatments. Significant differences were spotted within the same fungal species and the benzoquinone treatment regarding different days of exposure (Table 3). The inhibition halos decreased in diameter along the observation days, due to either the fading of the benzoquinones effect (e.g., due to benzoquinone chemical or fungal-induced degradation) and/or the adaptation of the fungus to benzoquinones exposure.

*Penicillium* sp. was the most affected fungal species, as the inhibition halo persisted after 10 days of exposure to B1 and to B2 (Table 3). On the other hand, *A. niger* recovered its development rapidly (as well as *Trichoderma* sp.). *A. flavus*, *A. fumigatus*, and *Fusarium* sp. exhibited an intermediate response, with a slower recovery when compared to *A. niger*. *Trichoderma* sp. exhibited a quick recovery after the B1 and B2 treatment. Interestingly, *A. flavus* showed a slower recovery from the B1 exposure than from the B2 treatment, in contrast to *Fusarium* sp. which produced an opposite response (Table 3).

### 3.2. Lethal Assays

The lethal assays were carried out using the fungal species that exhibited inhibition halos in the end of the disk diffusion assays; therefore, *Trichoderma* sp. and *Aspergillus* spp. were not submitted to lethality assays, as these fungi had a steady growth covering the full surface of the Petri dish, making the identification of the different growth zones (peripheral, transition, and central) impossible to determine.

The lethal assays were performed on *Fusarium* sp. exposed to B2 and *Penicillium* sp. exposed to both B1 and B2. The B2 treatments showed a fungistatic activity towards *Fusarium* sp. (Table 4), as after the isolation of mycelium from the different zones of the fungal colonies the fungus was capable of growth (Table 4). *Penicillium* sp. in contact with B1 showed a similar response but was unable to grow even after its isolation from the B2 treatment medium, indicating a putative fungicidal activity of B2 on this fungus.

## 4. Discussion

Benzoquinones treatments B1 and B2 showed an inhibitory, or fungistatic, effect on all six fungal species tested. At the minimum, a delayed mycelial growth was observed. Benzoquinones reduced the growth rate of all fungal species under study, although some species showed a quicker recovery of their growth rate, such as *Trichoderma* sp. and *A. niger*, which resumed their normal growth after the second and third days of exposure, respectively. Fungal species from the *Trichoderma* genus are suggested to be used as an efficient future alternative in the control of insect pests, due to their ability to produce insecticidal secondary metabolites, antifeedant compounds, and repellent metabolites; besides, they may perform direct parasitism of insects. All these abilities might be valuable for pest control in agricultural fields [37]. This might indicate that species from this fungal genus may have developed effective defenses against insects, namely tolerance to their putative defensive substances developed by insects.

Interestingly, *A. flavus* seemed to have a slower recovery from the B1 treatment than from the B2 treatment, although not significantly. In a previous study conducted by our team, mycotoxigenic *A. flavus* demonstrated a negative impact on *T. castaneum* when both organisms thrived on the same food resource, killing the insects despite their putative secretion of benzoquinones [38].

A lethal, or fungicidal, effect of the mixture of EBQ + MBQ and the inhibitory effect of BQ on *Penicillium* sp. were observed. *Penicillium* sp. was the most susceptible species to both benzoquinone treatments, especially to EBQ + MBQ (B2), which mimics the benzoquinone mixture secreted by *T. castaneum*, as it exerted a fungicidal effect on the *Penicillium* sp. mycelium close to the filter paper disk impregnated with B2 (Table 4). For *Fusarium* sp., an inhibitory effect of B2 is suggested, as this fungus grew after being isolated from the benzoquinone treatment. Fungi have different susceptibility levels to different organic compounds, depending not only on fungal species but also on the type and concentration of the compound [39,40]. The type of effect exerted by the bioactive compounds tested against the fungi is also dependent on their concentration, with inhibitory effects typically associated with lower concentrations in comparison to lethal effects [41].

The six fungal species studied and the insect *T. castaneum* forage for the same nutrient resources and flourish in identical abiotic conditions, suggesting that some sort of arms race or adaptations of both types of organisms may have evolved [20]. The presence of *T. castaneum* in maize flour may exert positive or negative effects on different fungal species [42]. The type of interaction may be synergistic or of competitive antagonism, or even both, depending on the population levels of both organisms. Dunkel [43] stated that some fungal species associated with stored products may increase insect populations, whereas other fungal species may repel or even secrete harmful toxins to the insects. It is also important to note that *T. castaneum* is able to feed on several fungal species, including *A. niger* or *Fusarium* sp., for example [44]. Fungi may also pose different kinds of threats to insect development and survival, and it has already been proven that some substances secreted by insects may have a defensive role against entomopathogenic fungi [20,45].

Fungi may take direct advantage of *T. castaneum* activity [38,42,46,47], with negative effects at the level of the insects’ fitness. Therefore, the insects’ defense mechanisms may have evolved to release defensive substances, such as the benzoquinones, which may have a regulatory effect on some fungal species (and other organisms), preventing their development, that would otherwise cause insects’ death [20]. The diversity of responses of the fungal species tested may also be linked to the presence of mechanisms of degradation of organic compounds such as benzoquinones. On the other hand, some fungi (for example, belonging to genera *Aspergillus*, *Fusarium*, or *Penicillium*) may produce volatile organic compounds, such as 1-octen-3-ol, with a direct insecticidal and/or repellent effect on *T. castaneum*, acting also as oviposition deterrents for *Sitophilus zeamais* (Motschulsky) [48].

This study shows that some fungal species are susceptible to benzoquinones secreted by *T. castaneum*. The benzoquinones used in this work were not directly extracted from *T. castaneum*, which would include a richer chemical mixture, with some other components besides BQ, EBQ, and MBQ [16,42]. In the future, the use of natural benzoquinones and/or the insects in these studies will be valuable to further investigate the nature of the interactions between *T. castaneum* and fungi present in stored maize. Additionally, testing the effect of different benzoquinones and fungal spore concentrations will add valuable information to the study of the interactions between these organisms. Unveiling the nature of these interactions may be timely due to arising concerns regarding the spread of both mycotoxigenic fungi and *T. castaneum* to new geographical areas due to climate-change scenarios [49,50,51].

## 5. Conclusions

Considering these results and previous studies by other authors, *T. castaneum* should be regarded not only as an important pest, but also, from a holistic point of view, as something integrated into the complex ecosystem of stored products and their associated organisms. This interaction between this insect and different species of fungi varies with the fungal species and population levels of both types of organism. The authors propose future studies where natural benzoquinone mixtures are used. The revelation of the nature of the relationship between selected fungal species and adults of *T. castaneum* can be a step towards better management in the protection of stored products.

## Figures and Tables

**Table 1:** Quantity (μg) and final concentration (μg/μL) of benzoquinones (B1: 1,4-benzoquinone (BQ) and B2: ethyl-1,4- benzoquinone (EBQ) plus methyl-1,4-benzoquinone (MBQ)) used in the disk diffusion assays.

		*T. castaneum* Adult (μg) n = 1	*T. castaneum* Adult (μg) n = 200	Final Concentration (μg/μL)
**B1**	BQ	45	9000	300
**B2**	EBQ	27	5400	180
	MBQ	18	3600	120

**Table 2:** Spore concentrations (number of spores per mL) from the six fungal species used for disk diffusion assays: *Aspergillus flavus*, *A. fumigatus*, *A. niger*, *Fusarium* sp., *Penicillium* sp. and *Trichoderma* sp.

Fungal Species	Spores Concentration (n.º of Spores/mL)
*Aspergillus flavus*	1.1 × 10^6^
*fumigatus*	1.3 × 10^6^
*A. niger*	1.9 × 10^6^
*Fusarium* sp.	3.6 × 10^6^
*Penicillium* sp.	1.1 × 10^6^
*Trichoderma* sp.	2.8 × 10^6^

**Table 3:** Average diameter (± standard deviation) of the inhibition halo of the fungi species tested (*Aspergillus flavus*, *Aspergillus niger*, *Fusarium* sp., *Penicillium* sp., *Trichoderma* sp.) exposed to control, solvent control (DMSO) and benzoquinone treatments (BZQ): B1 and B2.

Fungi	*A. flavus*		*A. fumigatus*		*A. niger*		*Fusarium* sp.		*Penicillium* sp.		*Trichoderma* sp.	
BZQ	B1	B2	B1	B2	B1	B2	B1	B2	B1	B2	B1	B2
**Day 1**	4.8 ± 0.2 a	4.4 ± 0.2 a	5.5 ± 0.3 a	5.1 ± 0.2 a	3.4 ± 0.2 a	3.4 ± 0.2 a	5.3 ± 0.2 a	5.0 ± 0.3 a	5.0 ± 0.1 a	4.8 ± 0.2 a	4.7 ± 0.4 a	4.2 ± 0.2 a
**Day 2**	3.8 ± 0.2 b	3.2 ± 0.2 b	3.9 ± 0.3 b	3.7 ± 0.3 b	2.4 ± 0.5 b	2.6 ± 0.5 b	4.7 ± 0.2 b	4.4 ± 0.3 b	4.6 ± 0.1 b	4.4 ± 0.2 b	2.7 ± 0.3 b	2.6 ± 0.0 b
**Day 3**	2.8 ± 0.3 c	2.0 ± 0.7 c	2.9 ± 0.4 c	3.0 ± 0.3 c	1.8 ± 0.8 c	1.8 ± 0.8 c	4.0 ± 0.3 c	3.8 ± 0.4 c	4.2 ± 0.1 c	4.1 ± 0.3 bc	0.0 ± 0.0 c	0.0 ± 0.0 c
**Day 4**	1.9 ± 0.3 d	1.3 ± 0.5 d	2.3 ± 0.3 d	2.6 ± 0.2 d	0.0 ± 0.0 d	0.0 ± 0.0 d	3.1 ± 0.2 d	3.1 ± 0.6 d	4.0 ± 0.2 d	3.8 ± 0.5 cd		
**Day 5**	1.2 ± 0.6 e	0.0 ± 0.0 f	1.9 ± 0.5 e	2.1 ± 0.3 e			2.2 ± 0.3 e	2.3 ± 0.7 e	3.6 ± 0.4 e	3.7 ± 0.6 cd		
**Day 6**	0.0 ± 0.0 f	0.0 ± 0.0 f	0.0 ± 0.0 f	0.0 ± 0.0 f			0.8 ± 0.9 f	1.1 ± 1.2 fg	3.1 ± 0.2 f	3.4 ± 0.8 cd		
**Day 7**							0.2 ± 0.5 fg	0.5 ± 1.2 g	2.7 ± 0.3 g	3.2 ± 0.8 de		
**Day 8**							0.0 ± 0.0 g	0.4 ± 1.1 g	2.4 ± 0.3 gh	3.1 ± 0.9 de		
**Day 9**							0.0 ± 0.0 g	0.3 ± 0.9 g	2.3 ± 0.2 h	2.8 ± 0.7 e		
**Day 10**							0.0 ± 0.0 g	0.3 ± 0.9 g	2.3 ± 0.2 h	2.8 ± 0.7 e		

* different letters within the same column means that there are significant differences among different days of exposure (*p* < 0.05).

**Table 4:** Evaluation of the growth of the fungal species tested (*Fusarium* sp., and *Penicillium* sp.) after the disk diffusion assays with contact with benzoquinones (BZQ): B1 and B2 treatments. Three different contact zones were tested for each fungus: peripheral zone, transition zone, and central zone (closer to the benzoquinone source, as outlined in Figure 1). 1—Fungal growth; and 0—No fungal growth.

Fungal Species	BZQ	Peripheral Zone	Transition Zone	Central Zone
*Fusarium* sp.	B2	1	1	1
*Penicillium* sp.	B1	1	1	1
	B2	1	1	0

## Data Availability

Data is contained within the article.
